# Structural and Pharmacological Characterization of Novel Potent and Selective Monoclonal Antibody Antagonists of Glucose-dependent Insulinotropic Polypeptide Receptor

**DOI:** 10.1074/jbc.M112.426288

**Published:** 2013-05-20

**Authors:** Peter Ravn, Chaithanya Madhurantakam, Susan Kunze, Evelyn Matthews, Claire Priest, Siobhan O'Brien, Andie Collinson, Monika Papworth, Maria Fritsch-Fredin, Lutz Jermutus, Lambertus Benthem, Markus Gruetter, Ronald H. Jackson

**Affiliations:** From the ‡ Department of Antibody Discovery and Protein Engineering, MedImmune, Milstein Building, Granta Park, Cambridge CB21 6GH, United Kingdom,; the § Department of Biochemistry, University of Zurich, Winterthurerstrasse 190, 8057 Zurich, Switzerland,; ¶ Discovery Science, AstraZeneca R&D Alderley Park, Cheshire SK10 4GT, United Kingdom, and; ‖ Bioscience, AstraZeneca R&D Mölndal, HF1 S-431 83 Mölndal, Sweden

**Keywords:** Antibody Engineering, Crystal Structure, Diabetes, G-Protein-coupled Receptors (GPCR), Phage Display, Antagonist, GIP Receptor (GIPr), Glucose-dependent Insulinotropic Polypeptide (GIP), Incretin

## Abstract

Glucose-dependent insulinotropic polypeptide (GIP) is an endogenous hormonal factor (incretin) that, upon binding to its receptor (GIPr; a class B G-protein-coupled receptor), stimulates insulin secretion by beta cells in the pancreas. There has been a lack of potent inhibitors of the GIPr with prolonged *in vivo* exposure to support studies on GIP biology. Here we describe the generation of an antagonizing antibody to the GIPr, using phage and ribosome display libraries. Gipg013 is a specific competitive antagonist with equally high potencies to mouse, rat, dog, and human GIP receptors with a *K_i_* of 7 nm for the human GIPr. Gipg013 antagonizes the GIP receptor and inhibits GIP-induced insulin secretion *in vitro* and *in vivo*. A crystal structure of Gipg013 Fab in complex with the human GIPr extracellular domain (ECD) shows that the antibody binds through a series of hydrogen bonds from the complementarity-determining regions of Gipg013 Fab to the N-terminal α-helix of GIPr ECD as well as to residues around its highly conserved glucagon receptor subfamily recognition fold. The antibody epitope overlaps with the GIP binding site on the GIPr ECD, ensuring competitive antagonism of the receptor. This well characterized antagonizing antibody to the GIPr will be useful as a tool to further understand the biological roles of GIP.

## Teaser

**Background:** GIPr mediates insulin secretion upon GIP stimulation.

**Results:** Gipg013 is a highly specific and potent antagonist of GIPr with a fully characterized mode of action.

**Conclusion:** Gipg013 antagonizes GIPr *in vivo*, as exemplified by inhibition of GIP-induced insulin secretion.

**Significance:** This antagonizing antibody to the GIPr will be useful as a tool to further understand the biological roles of GIP.

## Introduction

Glucose-dependent insulinotropic peptide (GIP)^2^ is an incretin hormone released from intestinal K cells in response to food intake (1–3). GIP primarily circulates as a 42-amino acid peptide (GIP(1–42)) but is also present in a form lacking the C-terminal 12 amino acids (GIP(1–30)), which exerts very similar effects at β-cells (4). The receptor for GIP (GIPr) is expressed on pancreatic β-cells, where activation leads to insulin release (5). GIPr is also expressed in adipocytes, which respond to increased GIP levels with increased glucose uptake, fatty acid synthesis, and fatty acid incorporation into lipids within the adipocytes (6). Whereas the related incretin hormone GLP-1 has found widespread therapeutic use, the complex nature of GIP biology has meant that proposals for agonism (7) or antagonism of the GIPr (8) for the treatment of diabetes and obesity have yet to lead to therapeutic applications.

To understand the complex effects of GIP on plasma glucose and fat deposition, it would be desirable to have a specific potent antagonist with an extended half-life. To date, only low potency antagonists with a short half-life have been available. Truncated GIP peptide antagonists have been reported: GIP(3–42) (9), GIP(6–30) (10), and GIP(7–30), which has an IC_50_ of ∼100 nm in a cAMP generation assay (5).

The GIPr belongs to the glucagon receptor subfamily of class 2 (class B) GPCRs, a class that also includes the receptors for GLP1, glucagon, parathyroid hormone, calcitonin, corticotropin-releasing factor, and other therapeutically important peptide hormones (11). The isolation of neutralizing antibodies to GPCRs has proven difficult, due to the low proportion of the receptor exposed on the extracellular surface and the involvement of ligand binding to transmembrane regions in receptor activation. Only limited examples have been published to date (*e.g.* monoclonal antibodies to the class A receptors CXCR4 and S1P3, from mouse hybridomas (12, 13), and CCR5, from human scFv phage display libraries (14, 15)). For class B GPCRs, neutralizing monoclonal antibodies in complex with the glucagon receptor and neutralizing polyclonal antibodies to the GIPr have been reported (16–18). More recently, fully human monoclonal antibodies to glucagon and GLP1 receptors have been obtained by immunization of mice transgenic for human antibody genes (19, 20). Crystal structures of monoclonal antibodies in complex with the glucagon receptor have been reported (21). In this paper, we report an antagonist antibody derived from phage display libraries, Gipg013, that shows potent competitive neutralization of GIP activity at its receptor. Gipg013 should prove to be a useful tool for understanding the biological effects of GIP at the GIPr.

The crystal structure of GIP(1–42) in complex with the extracellular domain (ECD) of the GIPr demonstrated that the hormone binds in an α-helical conformation in a surface groove of the ECD largely through hydrophobic interactions (22). It has been proposed that the C-terminal part of GIP first interacts with the ECD, and this event then helps the binding of the N-terminal part of the peptide with the juxtamembrane region of the receptor and activation of the receptor. The binding of peptides to class B receptors shows some common structural features. Superimposition of the crystal structures of these class B GPCRs shows that the sandwich fold, consisting of an α-helix and two anti-parallel β-sheets linked by three disulfide bonds, is well conserved in the family, although sequence alignment shows less conservation (23). We have determined the crystal structure of the Gipg013 Fab in complex with the GIPr ECD and compared this with the structure for GIP in complex with the GIPr ECD.

## EXPERIMENTAL PROCEDURES

### 

#### 

##### GIPr ECD

The GIPr ECD with an N-terminal His_6_ and FLAG tag was expressed and purified as described previously (22) and biotinylated using EZ-link Sulfo-NHS-LC-Biotin (Perbio/Pierce, product no. 21335). Parthier (22) reported the *K_d_* of GIP(1–42) for GIPr ECD as 1.1 μm as measured by calorimetry. The GIPr ECD preparation used in selections and screening was validated by competition with the cell surface GIPr on HEK293 cells for binding to GIP in the cAMP assay. An IC_50_ of 7.0 μm was obtained.

##### Cell Culture

Stable cell lines expressing human, mouse, rat, and dog GIP receptor (HEK293 human GIPr, mouse GIPr, rat GIPr, and dog GIPr) were generated in HEK293 cells. In brief, HEK293 cells were transfected with the expression vector pIRESneo3 containing the full-length GIP receptor gene of each species. Cells were maintained in Dulbecco's modified Eagle's medium (DMEM) (Invitrogen) supplemented with 10% FBS and 0.8 mg/ml Geneticin (G418) (Invitrogen) at 37 °C in a humidified environment containing 5% CO_2_. Cells were seeded every 2–4 days at a density to achieve 3–4 × 10^7^ cells on the day of the assay. Cells were harvested using Accutase (PAA Laboratories GmbH), counted, and resuspended in a suitable volume of appropriate assay buffer to achieve correct cell density for either selection or assay procedures, as outlined below.

##### Phage Display Libraries

The combined spleen library (8.5 × 10^10^) has been described by Lloyd *et al.* (24) and was included in the phage selections reported here. A sublibrary of the combined spleen library, S5 (1.8 × 10^10^) was investigated separately and was applied as the source of V genes for the generation of a separate ribosome display library.

##### Construction of Naive PBL Phage Display Libraries

40-μg aliquots of the ribosome display PBL libraries were digested with NcoI and NotI to excise the scFv constructs and clone them into pCantab6 (25). The ligations were transformed into *Escherichia coli* TG1 by electroporation, yielding libraries of 6.0 × 10^8^ and 8.0 × 10^8^ cfu for the phage display PBLκ and phage display PBLλ libraries, respectively. 88 clones from each library were picked and sequenced to validate the quality of the libraries, revealing at least 75% functional clones.

##### Ribosome Display Libraries

Generation of PBL library has been described in detail previously (26). Here we have applied the two subsets (κ and λ) as individual libraries in the selections. Similar to the PBL library, the S5 library was generated by subcloning the variable genes from the phage display S5 and recombining into a new ribosome display library.

##### Display Library Selection

The phage display and ribosome display selections on soluble biotinylated GIPr ECD were carried out as described previously (26–31). In brief, the phage display selections were performed with biotinylated GIPr ECD at 200, 50, 25, and 10 nm in rounds 1–4, respectively, to increase selection pressure throughout the selection rounds. The ribosome display selections were performed with biotinylated GIPr ECD at 200, 200, 50, and 10 nm in rounds 1–4, respectively. To enable cell surface selections, the outputs from the fourth round of ribosome display selection were subcloned to phage display format, yielding libraries of 1.5 × 10^7^, 7.9 × 10^6^, and 1.1 × 10^7^ for PBLλ, PBLκ, and S5, respectively. The cell surface selections were performed on HEK293 cells transformed with either an empty control construct or a construct for overexpressing the human GIP receptor and were performed with all of the subcloned fourth round ribosome display libraries and the second round phage display library selection outputs. The selections were monitored by screening outputs for scFv binding to GIPr ECD in phage ELISA as described previously (30).

##### Screening for GIPr Antagonism in Cell-based Activity Assay

The cAMP HTRF assay was based upon the ability of an antibody to inhibit the GIP/GIPr interaction (acting as an antagonist) and thus decrease cellular production of cAMP. The resultant assay signal is inversely related to the levels of cAMP generated. The cAMP dynamic 2 kit (Cisbio) was used according to the manufacturer's recommendations.

For the high throughput screen of scFv peripreps (∼10,500 clones, evenly picked across selections), HEK293 GIPr cells were resuspended in cell medium (DMEM, 10% FBS, 1.6% G418), containing 0.5 mm 3-isobutyl-1-methylanthine (Sigma), and 5 μl of cells were incubated with 2.5 μl of periplasmic preparations in a 384-well plate for 30 min at room temperature. Following this, 2.5 μl of GIP(1–42) peptide at 4 pm (an EC_80_ concentration) (Bachem) was added to samples, which were incubated for a further 30 min in order to produce an agonist response.

For profiling experiments, cells were incubated with a 2.5-μl dilution series of purified IgG or scFv (11-point, half-logarithmic dilutions) (32). The IC_50_ values were calculated by non-linear regression curve fitting using GraphPad Prism®, version 5.01.

##### Schild Analysis

The assay was carried out as the cAMP HTRF assay outlined above, with the following adjustments to measure dose-response curves at different antibody concentrations. Briefly, HEK293 human GIPr cells were incubated with a dilution series of a Gipg013 IgG antagonist starting from 3.75 μm (final concentration) or with assay buffer only (2.5 μl). Following incubation for 5 min, a dilution series of agonist (GIP peptide) (2.5 μl) was added to each concentration of Gipg013 IgG, and incubated for a further 30 min. EC_50_ values calculated for each antibody concentration and dose ratios determined using GraphPad Prism®. Subsequently, Schild plots were analyzed to yield p*A*_2_.

##### Binding Kinetic Estimation by Reflectometric Interference Spectroscopy

The Octet RED system (ForteBio) was used to determine the equilibrium dissociation constant (*K_D_*). The assays were performed in PBS supplemented with 10× kinetics buffer, using a volume of 200 μl for all incubations. The Super Streptavidin biosensors were loaded with biotinylated GIPr ECD at 1.2 μg/ml. All of the immobilized metal affinity chromatography-purified scFvs were assayed at ∼0.5 μm (12.5 μg/ml). The kinetic data sets were fitted using 1:1 Langmuir binding using Octet RED software to yield dissociation constants.

##### Purification of scFv and IgG

scFv was expressed in *E. coli* and purified from periplasmic extracts by immobilized metal affinity chromatography as described previously (33). For IgG conversion, the variable genes were cloned into the pEU vectors and expressed and purified as described previously (25).

##### Receptor Ligand Binding Assay

Fluorescent microvolume assay technology (FMAT) was used for the receptor ligand binding assay. Gipg013 or isotype control antibody NIP228 (0.25 μg/ml final concentration) was prepared in 384-well plates (10 μl). To these, 0.4 μg/ml (final concentration) AlexaFluor 647-labeled goat anti-human IgG H+L (Invitrogen) (10 μl) was added.

Stably transfected cell lines overexpressing either human, dog, mouse, or rat receptors for glucagon, GLP-1, or GIP were harvested and resuspended in Hanks' balanced salt solution (Invitrogen) containing 0.1% BSA (Sigma) and added to the 384-well assay plate (5000 cells in 20 μl). Plates were incubated in the dark at room temperature for 3 h. Subsequently, plates were read on the FMAT^TM^ 8100 HTS System (Applied Biosystems), and FL1 readings were plotted using GraphPad Prism®, version 5.01.

##### Receptor Ligand Competition Assay

FMAT was used for the receptor ligand competition assay. Dilution series of the antibodies were prepared in 384-well plates (10 μl). To this, 0.5 nm AlexaFluor 647-labeled GIP (Cambridge Research Biochemicals) (20 μl) was added. HEK293 human GIPr cells were harvested and resuspended in Hanks' balanced salt solution (Invitrogen) containing 0.1% BSA (Sigma), and added to the plate (10 μl). Plates were incubated in the dark at room temperature for 1–2 h. Subsequently, plates were read on the FMAT^TM^ 8100 HTS system (Applied Biosystems). For FMAT analysis, IC_50_ values were determined by non-linear regression curve fitting using GraphPad Prism®, version 5.01.

##### Binding Kinetics by Surface Plasmon Resonance

Real-time binding kinetics were analyzed by surface plasmon resonance using a Biacore 2000 Instrument. All reagents were purchased from BIAcore (Uppsala, Sweden). Experiments were carried out at 25 °C using a constant flow rate (30 μl/min) in running buffer (HBS-N; 10 mm HEPES, pH 7.4, 150 mm sodium chloride). Gipg013 IgG was immobilized on the sensor chip (CM5) at a low concentration using the amine coupling method as described by the manufacturer. Binding of GIPr ECD was observed at a range of concentrations (500, 250, 125, and 62.5 nm (concentrations used for the IgG experiment were 125, 62.5, 31.25, and 15.6 nm)). The sensor chip was regenerated with Biacore Regeneration Buffer (10 nm glycine-HCl, pH 1.5). Sensorgrams were analyzed using BIAevaluation software, version 3.0, using a 1:1 Langmuir binding model.

##### Crystallization, X-ray Data Collection, and Structure Solution

Gipg013 Fab (3.9 mg/ml) and GIPr ECD (0.77 mg/ml) protein samples were mixed at a 1:1 molar ratio in 0.1 m Tris buffer (0.1 m Tris, pH 7.6, 0.1 m NaCl). Using sparse matrix screens from Hampton Research and Molecular Dimensions (Suffolk, UK) in 96-well Corning plates (Corning Inc.) at 4 and 20 °C, preliminary crystallization conditions were identified. Sitting drop vapor diffusion experiments were performed using a Phoenix crystallization robot (Art Robbins Instruments). Gip013 Fab-GIPr ECD complex solution was mixed with reservoir solutions at a 1:1, 1:2, or 2:1 ratio (200-nl final volume), and the mixtures were equilibrated against 50 μl of reservoir solution. Crystallization conditions, data collection, and refinement statistics are summarized in Table 2. Crystals were flash-frozen in liquid nitrogen by adding 20% glycerol to the reservoir solution. Diffracting quality crystals were obtained from 0.02 m TAPS, pH 9.0, 30% (w/v) PEG 10,000.

X-ray data were collected using a MAR-345dtb image plate detector (MAR Research, Hamburg, Germany) mounted on a rotating anode x-ray generator equipped with a Helios optical system (Microstar Generator, Bruker AXS). Data were processed to 3.0 Å, using the programs MOSFLM (34) and SCALA (35). The complex structure was solved by molecular replacement using the program PHASER (36). Polyalanine models of incretin-bound extracellular domain of a GPCR (PDB code 2QKH) and murine IGG1 λ antibody (PDB code 1GIG) were used for GIPr ECD and Gipg013 Fab fragment, respectively. Refinement was done using the programs PHENIX-Refine (36), REFMAC5 (37), and COOT (38) with 5% of data set aside to calculate *R*_free_. Density modification was performed using DM in the program suite CCP4 followed by refinement, applying TLS refinement. The final structure was validated using the program PROCHECK (39). Figures were prepared using the program PyMOL (40).

##### Static Insulin Secretion Assays in Dispersed Rat Islets

Following isolation, islets were cultured in 11 mm glucose RPMI overnight. Subsequently, islets were collected and allowed to sediment, medium was removed, 1 ml of TryplExpress (Invitrogen) was added, and the islets were dispersed for 5 min. The cells were washed and reconstituted in KRH (129 mm NaCl, 5.0 mm NaHCO_3_, 4.8 mm KCl, 1.2 mm KH_2_PO_4_, 1.2 mm MgSO_4_, 10 mm Hepes, 2.5 mm CaCl_2_, 0.1% BSA fraction V, 3 mm glucose). The cell density was adjusted to 1.43 × 10^4^ cells/ml prior to incubation at 37 °C in 5% CO_2_. After 30 min, buffer was removed, and fresh KRH buffer was added to obtain the same density of cells, and 70 μl (1000 cells)/well was dispensed on a 96-well plate containing treatments, which was incubated at 37 °C in 5% CO_2_ for 60 min.

For the insulin quantification, the buffer was removed, and insulin was quantified using the insulin HTRF kit (CisBio). All samples were tested undiluted or diluted 1:2 on the low range in 384-well plates. All plates were read on an Envision plate reader. The raw data obtained from the insulin HTRF were expressed as ng/1000 cells/h. Results for each treatment group (*n* = 6) were averaged in Excel, S.E. was calculated.

##### Single Dose Pharmacokinetics Study with Gipg013 in Mice

Three groups of six female C57 mice were administrated Gipg013 at 3 mg/kg intravenously and 3 and 30 mg/kg Gipg0113 subcutaneously, respectively. Pharmacokinetics analysis was performed in a sandwich ELISA-based assay using mouse anti-human IgG, clone JDC-10 (Southern Biotech) for capture and HRP anti-human IgG, clone G18-145 (BD Pharmingen) for detection.

##### GIP-induced Insulin Secretion

Male Sprague-Dawley rats (350–480 g) were fasted for 6 h and anesthetized with Inactin® (120 mg/kg intraperitoneally). Body temperature was monitored with a rectal probe and maintained between 37.5 and 38.0 °C throughout the experiment. Animals were tracheotomized (polyethylene tubing PE 240), and catheters were placed in the right jugular vein (two PE10; one for substance administration and one for GIP delivery) and left carotid artery (PE50) for blood sampling.

The rats were given two identical 10-min periods of GIP infusion (50 pmol/kg/min) with 60 min between the periods. During each period, blood samples for insulin levels were collected at 0 (immediately before the start of GIP infusion) and 5, 10, 15, and 30 min after the start of GIP infusion. Vehicle (0.9% NaCl + 0.2% BSA, *n* = 6) or GIP013 (3 mg/kg or 30 mg/kg, *n* = 3) was administered as a bolus 30 min prior to start of the second GIP infusion. A blood sample was also collected 30 min prior to the first GIP infusion to obtain basal levels of glucose and insulin and 90 min after GIP013 infusion to determine substance plasma concentration. Insulin concentrations were measured using radioimmunoassay (rat insulin RIA kit; Linco Research, St. Charles, MO). The experiment was approved by the local ethics committee in Gothenburg, Sweden.

## RESULTS

### 

#### 

##### Selection of GIPr-antagonizing Antibodies

In order to generate antibodies specifically antagonizing the GIPr, scFv ribosome display and phage display libraries were selected for binding to purified GIPr ECD either as the sole enrichment method or followed by a selection step on GIPr-overexpressing cells. Selections were monitored for enrichment of clones binding to GIPr ECD and diversity. This identified 490 unique scFv clones binding to GIPr ECD. Phage and ribosome display selections generated populations of antibodies with different sequences, thereby supplementing each other in the generation of a large panel of binding clones (data not shown). Subsequently, selection outputs were screened for antibodies able to antagonize cAMP production, stimulated by the ligand GIP in a cell-based activity assay. The high throughput screen for inhibition of GIP-stimulated cAMP production identified 291 potential hits, of which 33 unique scFvs were confirmed in the profiling assay using purified scFv antibodies, with 23 clones showing full antagonism of the GIPr (IC_50_ values in the range of 4–178 nm). Binding kinetics of the scFvs to the GIPr ECD were investigated by reflectometric interference spectroscopy using an Octet RED instrument. Binding kinetics were estimated from a kinetic experiment at a single antibody concentration to provide a crude ranking of the clones, which displayed dissociation constants between 6 and 700 nm.

##### Conversion of Antibodies to IgG1 Format

**FIGURE 1.**
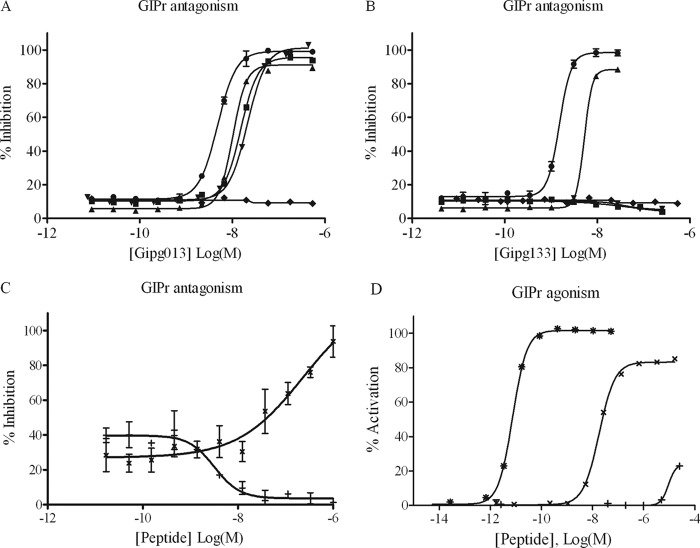
**Antagonism of GIP-induced cAMP production in GIPr-overexpressing cell lines.** Gipg013 and Gipg133 were characterized for GIPr antagonism in a cell-based cAMP HTRF assay, and data were plotted using nonlinear regression. *A* and *B* show antagonistic profiles from HEK293 cells overexpressing human, mouse, rat, and dog GIPr using Gipg013 and Gipg133 IgGs, respectively. *C* and *D* show antagonistic and agonistic profiles in the human GIPr assay for GIP(7–30), Pro3GIP, and GIP. Values have been normalized to the maximum activity of GIPr, which is defined by total cellular cAMP produced in the agonism assay or in the absence of peptide/IgG in the antagonism assay. Values shown are the mean ± S.E. (*error bars*) from duplicate wells, and data shown are representative of at least three separate experiments. Values shown are the mean ± S.E. from duplicate wells. ●, human; ■, mouse; ▾, rat; ▴, dog; ♦, isotype control IgG1; *, GIP; +, GIP(7–30); ×, Pro3GIP.

The VH and VL genes from the scFv clones were incorporated into constructs for full-length antibodies in the IgG1 format and expressed in mammalian cells. Purified IgGs were reprofiled in the cell-based activity assay to yield IC_50_ values for the antagonism of the human GIPr. Two clones retained full antagonist activity, whereas several clones lost potency or were characterized as partial antagonists. The antibodies Gipg013 and Gipg133 yielded complete antagonistic profiles with IC_50_ values of 6 and 2 nm, respectively (Fig. 1, *A* and *B*). Upon IgG conversion, Gipg013 gained potency from 233 nm as scFv to 6 nm as IgG, whereas Gipg133 only showed a modest gain in potency from 9 nm as scFv to 2 nm as IgG. The amino sequence of the variable domains of the two antibodies is shown in Table 1.

**TABLE 1:**
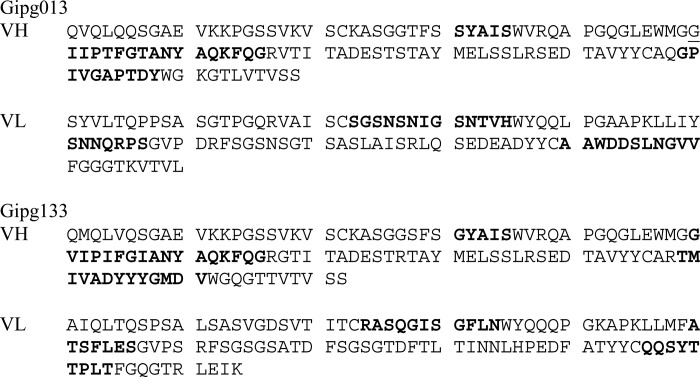
**Sequence of Gipg013 and Gipg133** Protein sequence of VH and VL with the CDR sequences shown in boldface type.

##### Antagonism of Mouse, Rat, and Dog GIPr

To evaluate species cross reactivity, Gipg013 and Gipg133 IgGs were profiled in the cAMP HTRF assay using HEK293 cells overexpressing either mouse, rat, or dog GIPr. Gipg013 IgG was found to cross-react with all three receptor species, with IC_50_ values ranging from 8 to 19 nm. Gipg133 IgG antagonized the dog receptor with an IC_50_ of 5 nm, but it had no effect on either mouse or rat receptors (Fig. 1, *A* and *B*) and is likely to bind to a different epitope on GIPr than Gipg013.

##### Comparing Properties of Antibodies with Other GIP Antagonists

Gipg013 and Gipg133 were compared with GIP(7–30) as an exemplar of antagonists derived by truncation of GIP. GIP(7–30) showed a much weaker antagonism of the receptor with an IC_50_ of 230 nm in our cell-based activity assay (Fig. 1*C*). The data are consistent with the IC_50_ of 100 nm reported by Tseng *et al.* (5) using a rat GIPr cell line. Pro3GIP did not antagonize the GIP-induced cAMP response in our activity assay (Fig. 1*C*), whereas antagonism by Pro3GIP was reported by Gault *et al.* (41) in an assay under significantly different conditions (100 pm GIP rather than 1 pm as in our study). In contrast, Pro3GIP partially agonized the receptor in our assay, to 83% of the response with GIP, with an EC_50_ of 180 nm (Fig. 1*D*).

**FIGURE 2.**
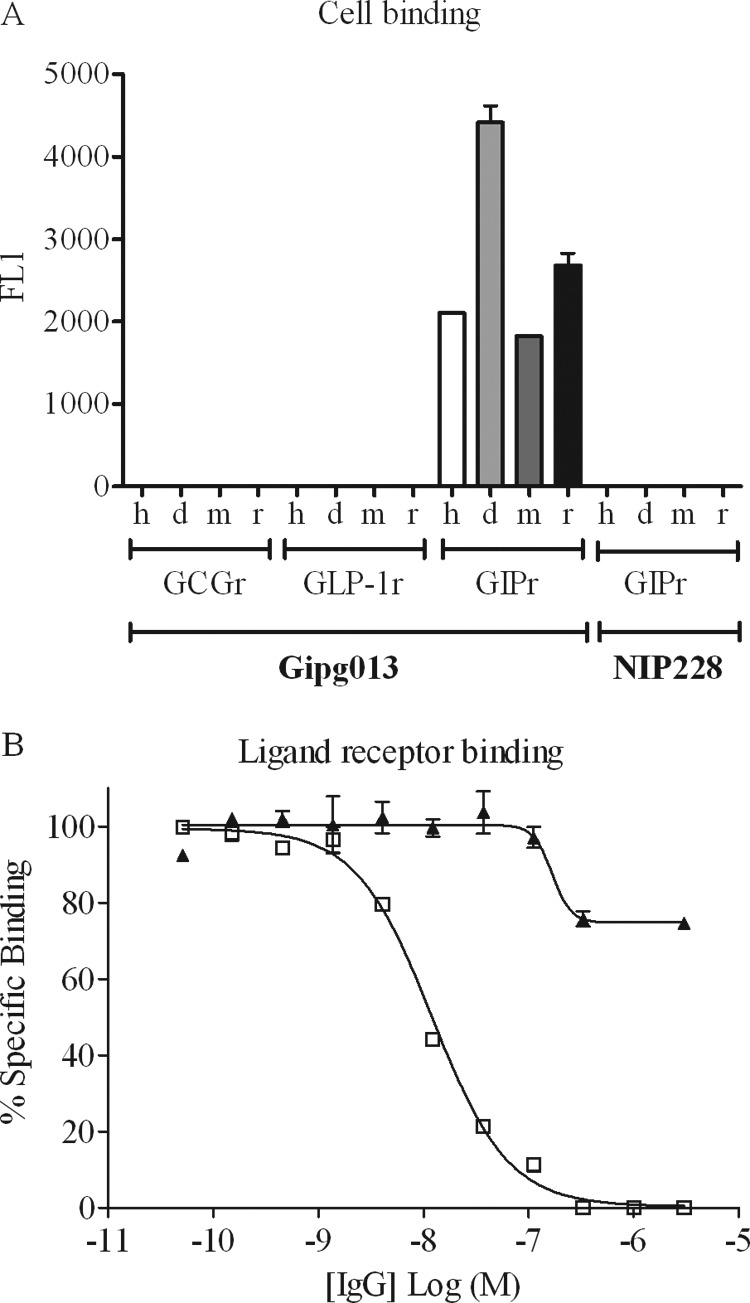
**Antibody cell binding and inhibition of ligand binding.** *A*, binding of Gipg013 to GIPr and related receptors on overexpressing cells. Shown is direct binding of 0.25 μg/ml Gipg013 IgG to human (*h*), dog (*d*), mouse (*m*), or rat (*r*) orthologs of GCGr, GLP-1r, or GIPr. Control for nonspecific binding on GIPr orthologs was NIP228_TM at 0.25 μg/ml. Values shown are the mean ± S.E. from duplicate wells, and data shown are representative of two separate experiments for the human receptors and a single experiment for the rodent and canine receptors. *B*, receptor ligand competition assay showing IC_50_ determination of Gipg013 IgG binding to GIPr-overexpressing cells. □, Gipg013; ▴, isotype control IgG1. Values shown are the mean ± S.E. (*error bars*) from duplicate wells, and data shown are representative of four separate experiments.

The specificity of the interaction of Gipg013 with the GIPr was assessed in FMAT cell binding assays using cell lines overexpressing human, mouse, rat, and dog GIPr or the related receptors GLP-1r and GCGr (Fig. 2*A*). As expected, binding was observed to the GIPr cell lines, with no detectable binding to any of the other cell lines.

##### Receptor Ligand Competition Assay with Gipg013

The competitive binding of Gipg013 to the GIPr was explored further in a receptor ligand competition assay. The IC_50_ for Gipg013 IgG was determined by competing a fixed concentration (below IC_50_ for GIP) of an AlexaFluor 647-labeled GIP peptide with increasing levels of the IgG antibody for binding to GIPr-overexpressing cells in an FMAT assay. Gipg013 IgG was shown to have an IC_50_ of 17.2 ± 6 nm for displacement of GIP from human GIPr-overexpressing cells (Fig. 2*B*). In comparison, the ligand GIP and the peptide antagonist GIP(7–30) gave IC_50_ values of 4.0 and 83 nm, respectively (data not shown), in good agreement with the 7 and 200 nm reported by Tseng *et al.* (5) using displacement of ^125^I-GIP bound to L293 cells.

##### Characterization of GIPr Antagonism by Gipg013

**FIGURE 3.**
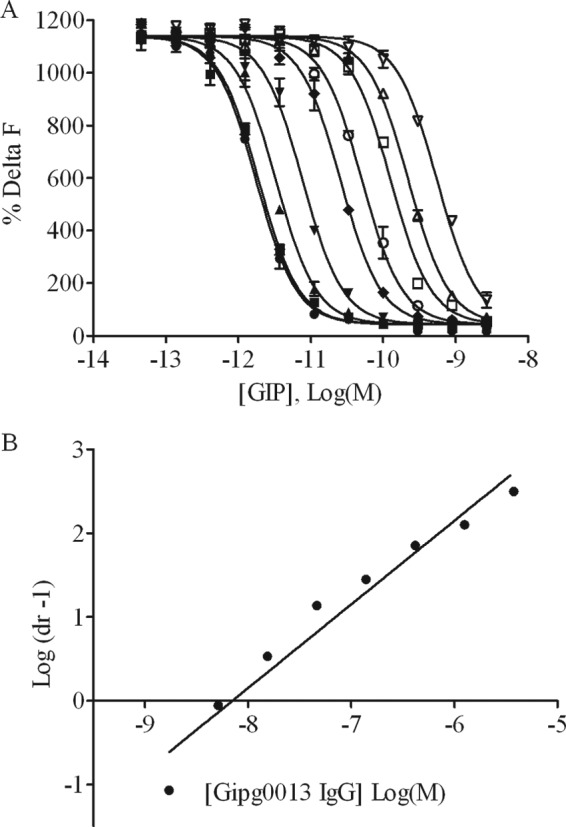
**Analysis of Gipg013 antagonism of GIPr.** *A*, GIP dose-response curves in the presence of Gipr013. The nonlinear regression plot of the dose-effect curve for GIP was determined in the presence of various concentrations of Gipg013: 3750 nm (▿), 1250 nm (▵), 417 nm (□), 139 nm (○), 46 nm (♦), 15 nm (▾), 5 nm (▴), 1.7 nm (■), and 0 nm (●). *B*, Schild plot analysis of dose-response curves. The Schild plot intersects the *abscissa* at p*A*_2_ (= *K_D_*). Values shown are the mean ± S.E. (*error bars*) from duplicate wells, and data shown are representative of three separate experiments.

Production of cAMP was measured as a function of GIP concentration in the absence or presence of a range of fixed Gipg013 concentrations. The dose-response curves as a function of GIP concentrations (Fig. 3*A*) were used for Schild regression analysis based on the EC_50_ values (Fig. 3*B*). The slope of the Schild plot (0.99 ± 0.08) confirmed that the antibody is competitive with the ligand GIP on the receptor. p*A*_2_ analysis of the Schild plot yielded a *K_d_* of 6.8 ± 0.6 nm.

##### Binding Kinetics of GipG013 Binding to Gipr ECD

The binding kinetics of the Gipg013 IgG were characterized in detail using surface plasmon resonance on a Biacore instrument. In contrast to the *K_d_* determinations with the Octet instrument, analysis was performed with the antibody immobilized on the Biacore chip and the GIPr ECD in solution. The IgG was immobilized at a low concentration (∼139 resonance units), and the binding interaction was analyzed at six different concentrations of GIPr ECD. The data gave a good fit when processed assuming a 1:1 Langmuir binding interaction. The kinetic analysis yielded association and dissociation rates of 1.59 × 10^5^ m^−1^ s^−1^ and 3.69 × 10^−3^ s^−1^, respectively, and a *K_d_* of 23 nm, which is comparable with the values of 7.45 × 10^4^ m^−1^ s^−1^, 1.88 × 10^−3^ s^−1^, and 25 nm measured for the Gipg013 scFv by reflectometric interference spectroscopy on the Octet RED. There was no dependence on whether the antibody was immobilized, as in surface plasmon resonance, or the GIPr ECD was immobilized, as for reflective interference spectroscopy. Therefore, the binding of the Gipg013 IgG to the GIPr does not seem to have a pronounced avidity effect. The difference in antagonistic potency between the scFv (IC_50_ = 233 nm) and the IgG (IC_50_ = 6 nm) could be caused mainly by steric effects.

##### Generation and Characterization of Gipg013 Fab

For the crystallization of Gipg013 with the GIPr ECD, we generated the Fab fragment of the antibody by papain digestion. As expected, the Gipg013 Fab retained binding to GIPr ECD, with a *K_d_* of 40 nm measured using BIAcore, and its ability to antagonize GIPr in our cell-based activity assay, with an IC_50_ of 102 nm, comparable with the *K_d_* of 25 nm and IC_50_ of 233 nm for the scFv, respectively.

##### Overall Crystal Structure

The Gipg013 Fab-GIPr ECD complex crystallized in space group *P2*_1_, and the structure was refined to a final *R*_cryst_ and *R*_free_ of 25.5 and 31.1%, respectively. The asymmetric unit contains two complexes, of which complex 1 (designated A-PQ, where A represents the GIPr ECD, and P and Q are the heavy and light chains of Gipg013 Fab, respectively) has an average temperature factor of 51.17 (〈*B_A_*〉) for the GIPr ECD as compared with complex 2 (designated B-CD, where B represents the GIPr ECD, and C and D are the heavy and light chains of Gipg013 Fab, respectively), where GIPr ECD has an average temperature factor of 94.57 (〈*B_B_*〉). Initial phasing resulted in only complex 1. After several refinement cycles, GIPr ECD in complex 2 could be modeled manually. Considering this factor, the remaining results and discussion involve complex 1 unless stated otherwise. Final refinement parameters are summarized in Table 2.

**TABLE 2:** **Data processing and refinement statistics**

**Unit cell parameters**	
*a*, *b*, and *c* (Å)	*a* = 48.3, *b* = 109.9, *c* = 105.9
α, β, and γ (degrees)	α = 90.0, β = 97.8, γ = 90.0
Space group:	*P2*_1_
Resolution range	47.71–3.0 Å
No. of molecules/asymmetric unit	2

**No. of reflections**	
Observed	90,279 (13,110)
Unique	21,867 (3167)
*I*/σ (*I*)	7.8 (2.6)
Completeness (%)	100.0 (100.0)
*R*_merge_ (%)*^a^*	16.0 (61.0)
Multiplicity	4.1 (4.1)
*R*_cryst_ (%)*^b^*	25.5
*R*_free_ (%)*^b^*	31.1
No. of protein atoms	7639
No. of chains	6

**Root mean square deviation from ideal geometry**	
Bond length (Å)	0.005
Bond angles (degrees)	1.150

***B* values (Å^2^)**	
Wilson *B*	48.89
Average *B*	48.22

**Ramachandran plot (%)**	
Residues in preferred regions	89.31
Residues allowed regions	10.58
Residues in disallowed regions	0.1

*^a^ R*_merge_ = Σ*_hkl_*Σ*_i_*‖*I_i_*(*hkl*) − (*I*(*hkl*))‖/Σ*_hkl_*Σ*_i_I_i_*(*hkl*), where *I_i_*(*hkl*) is the *i*th observation of reflection *hkl* and (*I*(*hkl*)) is the weighted average intensity for all observations *i* of reflection *hkl*. Values in parentheses refer to the highest resolution shell.

*^b^ R*_cryst_ and *R*_free_ = (Σ‖*F_o_*| − |*F_c_*‖)/(Σ|*F_o_*|), where |*F_o_*| is the observed structure factor amplitude and |*F_c_*| is the calculated structure factor amplitude.

##### Crystal Structure of Gipg013 Fab in Complex with GIPr ECD

**FIGURE 4.**
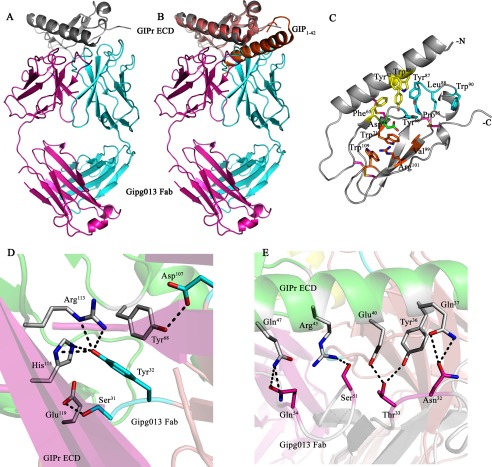
**Crystal structure of GIPr ECD and Gipg013 Fab complex.** *A*, overall structure of the complex where GIPr ECD and Gipg013 Fab heavy chain and light chains are represented in *gray*, *cyan*, and *magenta schematics*, respectively (PDB code 4HJ0). *B*, GIPr ECD-GIP(1–42) (PDB code 2QKH) superposed on GIPr ECD-Gipg013 Fab crystal structure. GIPr ECDs overlap with a root mean square deviation of 0.799 Å^2^. GIP(1–42) and GIPr ECD (2QKH) are shown in *orange* and *salmon schematics. C*, glucagon family recognition fold is highly conserved. The three clusters (*viz* cluster 1 (Trp^71^, Val^99^, Arg^101^, and Trp^109^ in *orange atomic color mode*), cluster 2 (Trp^39^, Tyr^42^, and Phe^65^ in *yellow atomic color mode*), and cluster 3 (Tyr^68^, Pro^85^, Tyr^87^, Leu^88^, and Trp^90^ in *cyan atomic color mode*)) are a characteristic feature of class B GPCR N-terminal extracellular domain. Asp^66^ in *green atomic color mode* is a highly conserved residue involved in stabilizing the structure. The three disulfide links are shown in *magenta atomic color mode. D*, CDRs of heavy chain play a vital role in complex formation. H-CDR1 (Tyr^32^) creates a network of hydrogen bond interactions with Arg^113^ and His^115^ of GIPr ECD. It is further aided by Ser^31^ (H-CDR1) interacting with Glu^119^ and Asp^107^ (H-CDR3) with Tyr^68^ of GIPr ECD. *E*, L-CDR1 and L-CDR2 of Gipg013 Fab light chain make a series of hydrogen bond interactions with N-terminal α-helix of GIPr ECD. In *D* and *E*, GIPr ECD, Gipg013 Fab heavy chain (H-CDRs) and light chains (L-CDRs) are represented in *white*, *cyan*, and *magenta atomic color modes*, respectively.

The crystal structure reveals the interface between the CDR loops of the Gipg013 Fab and the GIPr ECD (Fig. 4*A*). The overall structure of GIPr ECD is similar to the structure of the GIPr ECD in the GIPr ECD-GIP(1–42) complex (PDB code 2QKH) (21). The structures of the GIPr ECDs overlap with a root mean square deviation of 0.799 Å^2^ (Fig. 4*B*). The final model of Gipg013 Fab is composed of a heavy chain with 206 residues (Gln^3^–Val^217^) and a light chain with 211 residues (Ser^1^–Glu^213^) and shows the antibody binding site comprising residues from the complementarity-determining region loops outlined in Table 1.

##### The Glucagon Receptor Subfamily Fold of GIPr ECD

The GIPr ECD exhibits a three-layer α-β-βα fold, typical of the glucagon receptor subfamily of class B GPCRs (42), with three clusters of intramolecular interactions (Fig. 4*C*). GIPr ECD is a compact molecule with an N-terminal α_1_-helix (Ala^32^–Ala^52^) situated by the side of a central core created by two anti-parallel β-sheets. Further, each β-sheet comprises two β-strands (β1, Ser^64^–Phe^65^ and Cys^70^–Trp^71^; β2, Ala^78^–Ser^83^ and Phe^98^–Cys^103^). At the C-terminal end, two short helices, α2 (His^91^–Val^94^) and α3 (Thr^116^–Cys^118^), are present. The whole structure is stabilized by three disulfide links provided by Cys^46^–Cys^70^, Cys^61^–Cys^103^, and Cys^84^–Cys^118^ residues, which are a characteristic feature of the N-terminal domain of class B GPCRs (43) (Fig. 4*C*).

The overall fold of the GIPr ECD in complex with Gipg013 Fab correlates to the GIPr prototype described by Parthier *et al.* (22), but there are differences in detailed interactions. The glucagon family recognition fold in the Fab complex has the highly conserved aspartate (Asp^66^) at its center, creating backbone amide interactions with Tyr^68^ and Val^69^ unlike in the GIPr ECD-GIP(1–42) complex, where Asp^66^ has amide interactions with Met^67^, Tyr^68^, and Val^69^ (22). Furthermore, Asp^66^ provides stability to the GIPr ECD by side chain hydrogen bond interaction with -NH1 of Arg^113^ (bond length, 3.39 Å) and electrostatic interactions with Trp^71^. Asp^66^ does not form a salt bridge to Arg^101^ as observed in the case of CRFR-2β ECD (Asp^65^) (44).

##### Epitope of Gipg013 Fab on GIPr ECD

Gipg013 Fab binds GIPr ECD through a series of hydrogen bond interactions between the antibody CDRs and GIPr ECD, as shown in Table 3. The Gipg013 Fab heavy chain plays a crucial role in complex formation. The residue Tyr^32^ of H-CDR1 interacts with Arg^113^ and His^115^ of GIPr ECD through the side chain -OH. Furthermore, the interactions of Ser^31^ (H-CDR1) with Glu^119^ as well as Asp^107^ (H-CDR3; Asp^101^ in Kabat numbering) with Tyr^68^ stabilize the complex (Fig. 4*D*). The light chain CDRs further create stable interactions for the complex. L-CDR1 (Asn^32^ and Thr^33^) and L-CDR2 (Ser^51^ and Gln^54^) of Gipg013 Fab provide more hydrogen bond interactions to the N-terminal helix of GIPr ECD, as shown in Fig. 3*E*. The side chain of Asn^32^ (-Oδ1) interacts with the side chain of Gln^37^ (-Nϵ2) (GIPr ECD). In addition to this, Thr^33^ (-OH) interacts with the residues Tyr^36^ (-OH) and Glu^40^ (-Oϵ1) of GIPr ECD through side chain contacts.

**TABLE 3:**
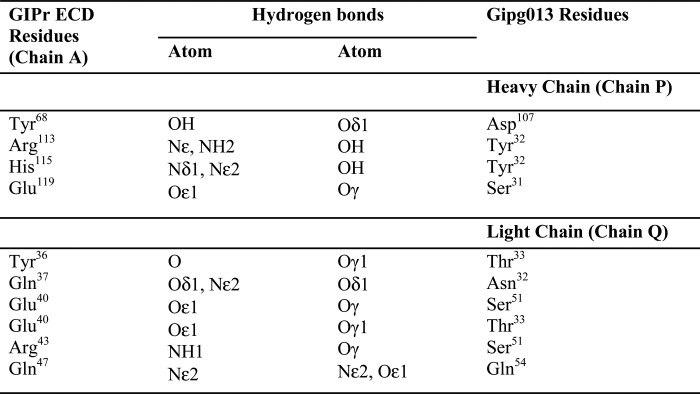
**Hydrogen bond interactions between GIPr ECD and Gipg013 Fab, complex 1 (A-PQ)** γ, δ, ϵ is the standard nomenclature for atom position on the amino acid.

From analysis of the accessible surface area and the buried surface area using the program PISA, a buried surface area of 5210 Å^2^ was calculated for the GIPr ECD-Gipg013 Fab complex. This includes ∼3100 Å^2^ of buried surface area at the interface of the Gipg013 Fab heavy and light chains. At the antibody binding site, there is a buried surface area of 1050 Å^2^ on the GIPr surface and 690 and 366 Å^2^ on the heavy and light chains of Gipg013 Fab, respectively, giving a total buried surface area of 2106 Å^2^. This contrasts with 1250 Å^2^ buried in the GIPr ECD-GIP(1–42) complex with 625 Å^2^ buried on each of the GIPr and GIP(1–42) surfaces. Thus, Gipg013 Fab has a large epitope on the GIPr ECD, binding to residues overlapping the binding site for the ligand GIP as well as to further residues burying a larger surface on the GIPr ECD. This confers higher stability on the GIPr ECD-Gipg013 Fab complex compared with the GIPr ECD-GIP(1–42) complex, as indicated by calculated free energy values (Table 4). Thus, Gipg013 Fab has a large epitope on the GIPr ECD, binding to residues overlapping the binding site for the ligand GIP as well as to further residues burying a larger surface on the GIPr ECD.

**TABLE 4:** **Comparison of the estimates of accessible surface area and the buried surface area in the GIPr ECD-Gipg013 Fab and GIPr ECD-GIP(1–42)_complexes using PISA_**

Complex	Accessible surface area	Buried surface area	Δ*G*^int*^a^*^	ΔG^diss*^b^*^
	Å*^2^*	Å*^2^*	*kcal/m*	*kcal/m*
GIPr ECD- Gipg013 Fab, A-PQ*^c^*	23,740	5210	−34.0	4.0
GIPr ECD- Gipg013 Fab, B-CD*^c^*	24,300	5110	−39.5	7.1
GIPr ECD-GIP(1–42), PDB code 2QKH	7920	1250	−7.9	−0.3

*^a^* Solvation free energy gain upon formation of the assembly, in kcal/m.

*^b^* Free energy of assembly dissociation, in kcal/m, where Δ*G*^diss^ > 0 indicates a thermodynamically stable complex.

*^c^* A-PQ and B-CD denote the complex chains in the asymmetric unit of the crystal structure of GIPr ECD- Gipg013 Fab, where A and B are GIPr ECDs, and PQ and CD are Fab fragments of Gipg013. When the interface of heavy and light chains of Gipg013 Fab is excluded in the complex for free energy calculations, complex 1 (A-PQ) has a Δ*G*^int^ and Δ*G*^diss^ of −11.9 and 2.4, respectively. Complex 2 (B-CD) has a Δ*G*^int^ and Δ*G*^diss^ of −12.1 and 2.2, respectively, indicating a stable complex compared with GIPr ECD-GIP(1–42).

##### Inhibition of GIP Enhancement of Glucose-stimulated Insulin Secretion

**FIGURE 5.**
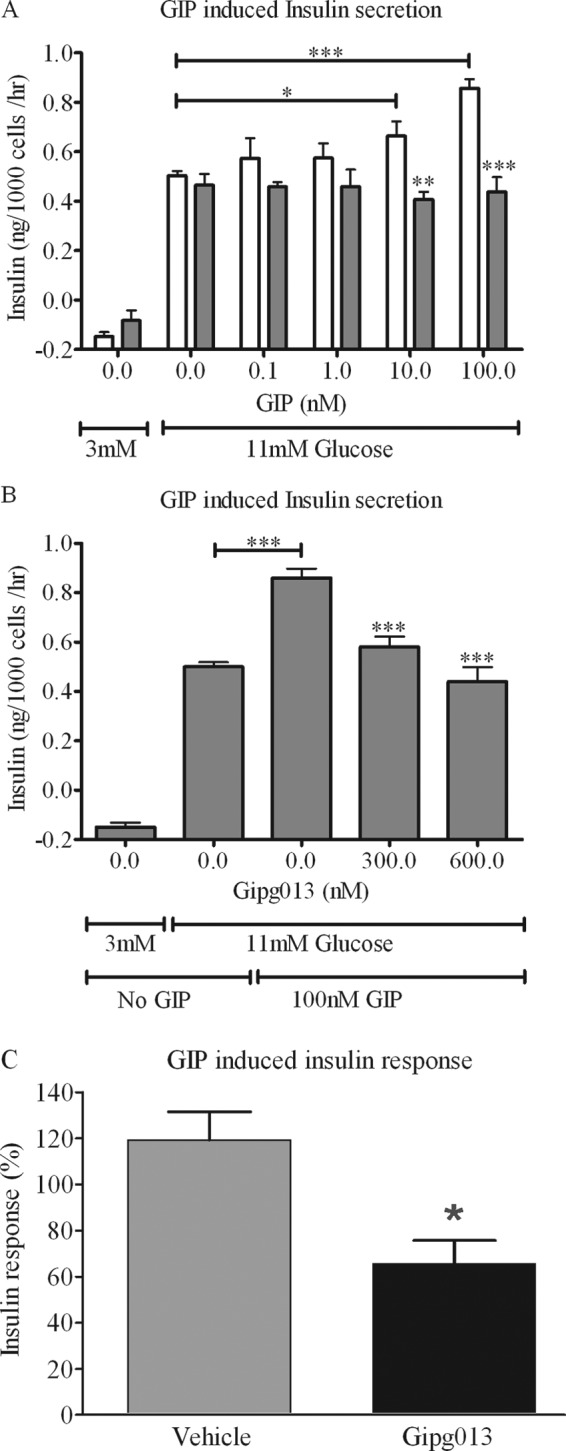
**Inhibition of GIP-induced insulin secretion.** *A* and *B* show inhibition of GIP stimulation in a glucose-stimulated insulin secretion assay with dispersed rat islets. *A*, effect of GIP on top of the glucose-stimulated insulin secretion (*open bars*) and the inhibition of this effect with 600 nm Gipg013 (*filled bars*). *B*, the GIP enhanced insulin secretion was inhibited with Gipg013 at concentrations of both 300 and 600 nm (*, *p* < 0.05; **, *p* < 0.01; ***, *p* < 0.001). *C*, inhibition of GIP-induced insulin secretion *in vivo*. Insulin response was measured as the relative insulin AUC_0–30 min_ between period 1 and 2 for GIP-induced insulin secretion in anesthetized rats, AUC1 before and AUC2 30 min after Gipg013 infusion. Insulin response = AUC2/AUC1 × 100. *n* = 6 for vehicle and *n* = 3 for Gipg013. *, *p* < 0.05 between vehicle and Gipg013. *Error bars*, S.E.

Inhibition of GIP enhancement of glucose-stimulated insulin secretion was studied in isolated rat pancreatic islet cells. GIP stimulated further the increase in insulin secretion in response to raising the glucose concentration from 3 to 11 nm, with greater enhancement as the GIP concentration increased to 100 nm. At all concentrations of GIP, this further stimulation by GIP was essentially abolished by the addition of the antibody Gipg013 (600 nm) (Fig. 5*A*). This suppressed GIP stimulation was also highly significant at 300 nm Gipg013 (Fig. 5*B*). In a separate experiment, there was an 81% reduction of GIP-induced insulin secretion at 200 nm Gipg013 (*p* < 0.004) compared with the no antibody sample, but at concentrations of Gipg013 of 60 nm or lower, the difference was not statistically significant (data not shown). Thus, Gipg013 inhibits GIP-induced insulin secretion in isolated pancreatic islet cells with increased effects at higher concentrations.

##### In Vivo Half-life of Gipg013

To demonstrate the extended half-life of Gipg013, the antibody was dosed to C57 mice for evaluation of pharmacokinetics. The half-life of antibody was ∼10 days, as expected for the human IgG1 format (45) and considerably longer than the half-life in the range of minutes predicted for GIP(7–30). The antibody was well tolerated, with no adverse events observed in a repeated dosing scheme of subcutaneous administration of 30 mg/kg every 5 days for 5 weeks. Serum samples were collected 99 h after the final dose, and an average antibody concentration of 5 μm was determined, in good agreement with the expected concentration from pharmacokinetic modeling. The antibody in the serum samples was active, with no loss of activity when compared with control serum added to 5 μm Gipg013 in both the ligand receptor competition assay and the cell-based activity assay (data not shown).

##### Inhibition of GIP-induced Insulin Secretion

Inhibition of GIP-induced insulin secretion by GIPr antagonism with Gipg013 was assessed in a rat infusion model. Prior to the experiment, it was established that the magnitude of the insulin response to a 10-min GIP infusion differed between animals but was similar when repeated in the same animal. Therefore, the first GIP infusion period served as the control to the second, Gipg013-treated period.

The area under the insulin response curve during 30 min from the start of the 10-min GIP infusion (AUC_0–30 min_) was significantly suppressed by Gipg013 (Fig. 5*C*). 3 and 30 mg/kg Gipg013 induced a similar suppression of insulin secretion, indicating that the maximum response was reached already at the lower dose, and therefore these doses were grouped together in the analysis. Average plasma concentration 90 min after dose was 97 and 1150 mg/liter (0.6 and 7 μm) for 3 and 30 mg/kg, respectively. The plasma levels of antibody at both doses were considerably higher than the total dose of GIP delivered to the mice during the infusion (500 pmol/kg, ∼15 nm). Thus, Gipg013 suppressed GIP-induced insulin secretion *in vivo*.

## DISCUSSION

In this study, we have generated a highly specific and potent antagonistic antibody against GIPr. The antibody Gipg013 is a competitive antagonist with respect to GIP-stimulated cAMP production at the receptor, giving a *K_d_* of 6.8 nm by Schild analysis. Gipg013, specifically antagonizes the GIPr, with no detectable inhibition of the related GLP1r and GCGr. It is equally potent on human, mouse, rat, and dog GIP receptors and will therefore be a useful pharmacological tool for elucidating biological effects at the GIPr in model systems. Insight into the mechanism of antagonism has been provided by the crystal structure of Gipg013 Fab bound to the extracellular domain of the GIPr. The epitope for the antibody overlaps with the docking site of the GIP peptide observed in the crystal structure of the complex of GIP with the GIPr ECD (22).

GIP binds the soluble GIPr ECD very weakly (IC_50_ ∼1 μm (22)) compared with the full-length receptor expressed in cells. Wheeler *et al.* (46) reported a *K_d_* of 200 pm for human GIP and an IC_50_ of 2.6 nm for displacement by human GIP of ^125^I-GIP from full-length GIP receptor expressed in CHO cells, similar to the IC_50_ of 4 nm for GIP displacement used in this study. The increased affinity for the full-length receptor arises from extra binding interactions as follows. GIP binds first to the ECD through its α-helical region between residues 12 and 30; the N-terminal region of GIP is then thought to interact with the membrane-associated portion of the GIPr, leading to stimulation of cAMP production (22). In contrast, the antibody binds exclusively to the GIPr ECD, showing equally high affinity to the free GIPr ECD and the cell-bound form.

**FIGURE 6.**
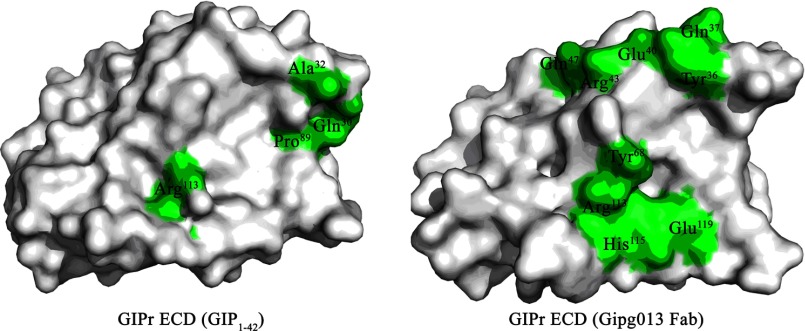
**Gipg013 Fab exhibits high affinity toward GIPr ECD.** Shown are footprints of GIP(1–42) and Gipg013 Fab on GIPr ECD domains. The buried surface area is greater in the GIPr ECD-Gipg013 Fab complex (see Table 4), and the interacting residues are labeled and shown on a *green shaded surface*. Only four residues are involved in hydrogen bond interactions with GIP(1–42).

There is a wide ranging footprint of Gipg013 Fab over GIPr ECD in the complex, leading to a larger buried surface area (Fig. 6). Several hydrogen bonds along with electrostatic interactions play a major role in forming the stable complex between Gipg013 Fab and GIPr ECD. In the case of the GIPr ECD-GIP(1–42) complex, Gln^30^, Ala^32^, Pro^89^, and Arg^113^ of GIPr ECD are involved in complex formation. On the other hand, the GIPr ECD-Gipg013 Fab complex is formed through interactions of Tyr^36^, Gln^37^, Glu^40^, Arg^43^, and Gln^47^ as well as Tyr^68^, Arg^113^, His^115^, and Glu^119^. This explains the higher affinity of the Gipg013 Fab toward GIPr ECD as compared with GIP(1–42).

Gipg013 does not show any detectable inhibition of or binding to GLP-1r and GCGr, which exhibit a sequence identity with GIPr ECD of 36% for GLP1r and 42% for GCGr. Comparison of the sequences of human GIPr, GLP1r, and GCGr with the epitope for Gipg013 Fab (Table 5*A*) not only reveals the basis for this selectivity but defines the critical interactions for the affinity of Gipg013 Fab for GIPr. Tyr^68^ and Arg^113^, which are important in complex formation with Gipg013 Fab, are highly conserved in all these human hormone receptor ECDs. Indeed, the homologous residues (Tyr^65^ and Arg^111^) contribute to the epitope of an antagonist antibody to the GCGr (21). However, although the majority of interacting residues are similar to GIPr, in GLP1r, valine replaces Tyr^36^ and leucine replaces His^115^. GIPr His^115^, which provides stronger hydrogen bond interactions through -Nδ1 and -Nϵ2 to the side chain of VH CDR1 Tyr^32^, may play a critical role in the affinity of Gipg013 Fab. Tyr^36^ of GIPr is required for the side chain interaction with Thr^33^ of VL CDR1, and replacement of Tyr^36^ with valine in GLP1r effectively removes the contact. Similarly, in GCGr, alanine and phenylalanine replace His^115^ and Tyr^36^, respectively, supporting the definition of these residues as crucial contacts for Gipg013 Fab. Multiple sequence alignment of human, dog, mouse, and rat GIPr (Table 5*B*) reveals that all of the residues involved in binding to the antibody are conserved between human and dog sequences, and seven of eight are conserved in mouse and rat sequences. The Gly for Arg^43^ substitution found in the mouse and rat GIPr and the human GCGr removes a hydrogen bond interaction with -Oγ of Ser^51^ through side chain atoms but has little or no effect on the antibody potency or binding. The antagonism by Gipg013 at GIPr of different species is therefore explained by the observed crystal structure contacts. The properties of Gipg013 IgG make it suitable for use in animal pharmacology and toxicology models.

**TABLE 5:**
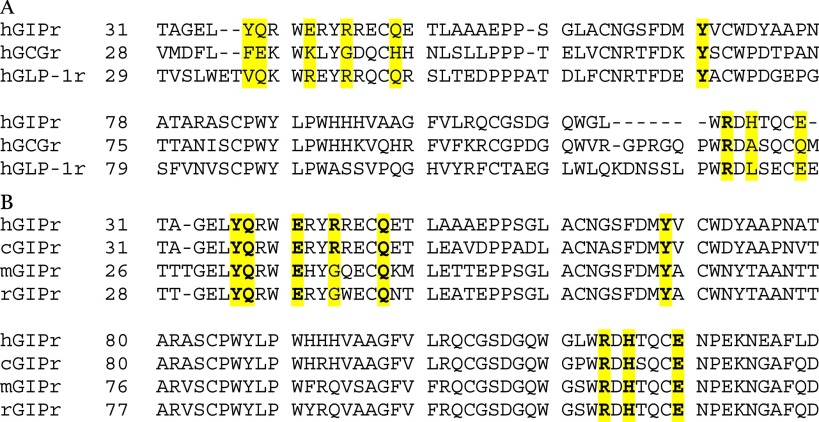
**Multiple sequence alignments** *A,* alignment of ECDs of human hormone receptors GIPr, GLP1r, and GCGr. The numbering is as per the crystal structure of GIPr ECD in complex with Gipg013 Fab. Highlighted residues are involved in the binding to Gipg013 Fab. Residues in boldface type are conserved residues. Protein sequences were obtained from the following Uniprot entries: human GIPr, P48546; human GCGr, P47871; human GLP-1r, P43220. *B,* alignment for comparison of contact residues for Gipg013 Fab in GIPr ECD of human, dog, mouse, and rat GIPr. The numbering is as per the crystal structure of GIPr ECD in complex with Gipg013 Fab. Highlighted residues are involved in the binding to Gipg013 Fab. Residues in boldface type are conserved residues. Protein sequences were obtained from the following Uniprot entries: human GIPr, P48546; canine GIPr, E2RIK5; mouse GIPr, Q0P543; rat GIPr, P43219.

The strategy used to generate Gipg013 favors the selection of antibodies that compete for ligand binding and should be generally applicable to the isolation of antagonistic antibodies against class B GPCRs, such as the GLP1 and glucagon receptors. It is proposed that the C-terminal helical portion of GIP first interacts with the GIPr ECD, and this event then helps the binding of the N-terminal part of the peptide with the juxtamembrane region of the receptor and activation of the receptor. Selection of antibody phage display libraries on purified receptor extracellular domain enriches for antibodies that bind to the same region of the receptor as the ligand. Further selection of the outputs on cells overexpressing the receptor enriches for antibodies that recognize the receptor in its native conformation and therefore antagonize GIP action in a native context. In previous studies in our laboratory where naive antibody phage display libraries have been directly selected only on GPCR-overexpressing cell lines, only modest enrichment of antibodies specific for receptors has been obtained, and consequently neutralizing antibodies remained rare in selection outputs. The selection on purified ECD will enrich for binders to the target and largely eliminate background usually associated with cell selections, thereby enriching primarily specific antibodies recognizing the native receptor. The complex steric effects involved in antagonism by the antibodies are indicated by the reduction in antagonistic activity for many antibodies when converting antibody clones from the monomeric scFv to the dimeric IgG format. Upon conversion, only two of the 30 clones retained full antagonist activity, including Gipg013. Several IgG antibodies were partial antagonists, whereas the rest lost all activity. This emphasizes the value of the strategy deployed here, where a large panel of clones was screened as IgG.

The biological role of GIP and the interaction with GIPr have been extensively studied, but uncertainty remains about the predicted therapeutic effects of modulating GIP action to regulate plasma glucose and fat deposition in adipocytes. The role in glucose regulation through insulin secretion suggests a therapeutic potential for GIPr agonism in type 2 diabetes (7). However, these patients are resistant to the insulinotropic effects of GIP (47). Transgenic mice overexpressing GIP exhibited reduced diet-induced obesity, although excessively elevated levels led to GIP resistance (48). Conversely, reduced body weight gain has been observed in diet-induced obesity models in a GIPr^−/−^ knock-out mouse (49), upon immunization with GIP to generate antagonistic antibodies, and upon administration of the GIPr peptide antagonist Pro3GIP or ablation of GIP-producing K cells (50–53).

However, the phenotypes observed cannot be definitively linked to the actions of GIP at its receptor. Alternative mechanisms and pathways may compensate for the ablation of the receptor in genetic knock-out models (54–56). Some antibodies generated by immunization could have the effect of extending the half-life of GIP in plasma by acting as a carrier, and this effect may overcome neutralization by other antibodies, as has been observed with MCP-1 (13). Furthermore, Pro3GIP, which has been proposed to antagonize GIPr (32), is an agonist of GIPr-mediated cAMP production in the assays reported in this study, consistent with the response noted by others (57). Finally, a small molecule antagonist of the GIPr, which has recently been reported to have an IC_50_ of 2.5 μm in a GIP-dependent cAMP assay, also retains some antagonistic activity at the glucagon receptor (58).

We suggest that a competitive antagonist at the receptor will more efficiently block signaling through the GIPr *in vivo*, especially because incretins show pulsative high intensity signaling. Gipg013 does indeed antagonize the GIPr and efficiently inhibit GIP-stimulated insulin secretion, as demonstrated in our rat *in vitro* and *in vivo* models. We have confirmed the extended half-life of Gipg013 and demonstrated antagonistic activity in serum after more than 4 days. The competitive mechanism, high potency, and selectivity combined with the extended half-life make Gipg013 an attractive antagonist of GIPr for future chronic pharmacological studies.

## Footnotes

2 The abbreviations used are:

GIP: glucose-dependent insulinotropic polypeptide
CDR: complementarity-determining region
FMAT: fluorescencent microvolume assay technology
GCGr: glucagon receptor
GIPr: GIP receptor
GLP-1r: GLP-1 receptor
ECD: extracellular domain
GPCR: G-protein-coupled receptor
HTRF: homogeneous time-resolved fluorescence
PBL: peripheral blood lymphocyte
scFv: single chain fragment variable
VH: variable fragment heavy chain
VL: variable fragment light chain
TAPS: 3-{[2-hydroxy-1,1-bis(hydroxymethyl)ethyl]amino}-1-propanesulfonic acid
PDB: Protein Data Bank.
